# A case report of serpentine-like syndrome and review of literature

**DOI:** 10.1186/s12884-022-04477-6

**Published:** 2022-03-04

**Authors:** Shijing Song, Jingjing Wang, Jijing Han, Yinghua Xuan, Wenxue Zhi, Qingqing Wu

**Affiliations:** 1 Ultrasound Department, Beijing Obstetrics and Gynecology Hospital, Capital Medical University, No.251 Yaojiayuan Road, Chaoyang district, Beijing, 100026 P. R. China; 2 Beijing Maternal and Child Health Care Hospital, Beijing, P. R. China

**Keywords:** Serpentine-like syndrome, Intrathoracic stomach, Brachioesophagus, Vertebral deformity, Case report

## Abstract

**Background:**

Serpentine-like syndrome (SLS) is a rare foetal abnormality, characterized by brachioesophagus, secondary intrathoracic stomach and vertebral deformity. Herein, we report a case of SLS diagnosed based on imaging, genetic examination and autopsy findings.

**Case presentation:**

From the 19th to 23rd weeks of gestation, the foetus presented with brachioesophagus, secondary intrathoracic stomach, intrathoracic spleen with poly-spleen malformation, spinal deformity and diaphragm dysplasia, and some abdominal organs were partly located in the thoracic cavity. After extensive counselling, the couple opted to terminate the pregnancy. Whole genome sequencing and autopsy were performed. Then, the foetus was diagnosed with SLS.

**Discussion and conclusions:**

SLS is characterized by multiorgan deformities and is associated with poor prognosis. Multiorgan deformities can be detected on prenatal sonography using three-dimensional ultrasound technology.

## Background

Serpentine-like syndrome (SLS) is a rare abnormality characterized by brachioesophagus, secondary intrathoracic stomach and vertebral anomalies, which are similar to the anatomical structures of snakes. To date, there are only nine reported cases of SLS, and most patients had a poor prognosis. Michael S. Katz et al. first discussed SLS in 2008 [1]. Obstetricians and neonatal paediatricians do not have sufficient knowledge about its characteristics. Hence, the actual number of SLS cases may be higher than that reported. In 2008, another case of SLS was described, and it was characterized by a congenital intrathoracic stomach, short oesophagus, hemivertebrae at the T5 level and complete gastric outlet obstruction [2].

Multiorgan abnormalities can be diagnosed prenatally via ultrasonography (US) and magnetic resonance imaging (MRI). US is advantageous as it is radiation-free and less time-consuming. Thus, it is considered an optimal examination method. However, due to the low spatial resolution of two-dimensional (2D) US, the whole lesion cannot be completely visualized. In addition, in SLS, some abnormalities cannot be directly diagnosed on 2D-US [3]. Advancements in three-dimensional (3D) US can provide opportunities for detecting multiorgan malformations. Hence, an accurate diagnosis, which is essential for adequate counselling and pregnancy management, can be obtained. Crystal Vue (Samsung Medison Co., Ltd., Seoul, South Korea) can improve the spatial resolution of ultrasonic images and can identify the important details of diseases. Hence, it can have a better diagnostic value in detecting multiple organ malformations [4].

## Case presentation

**Fig. 1:**
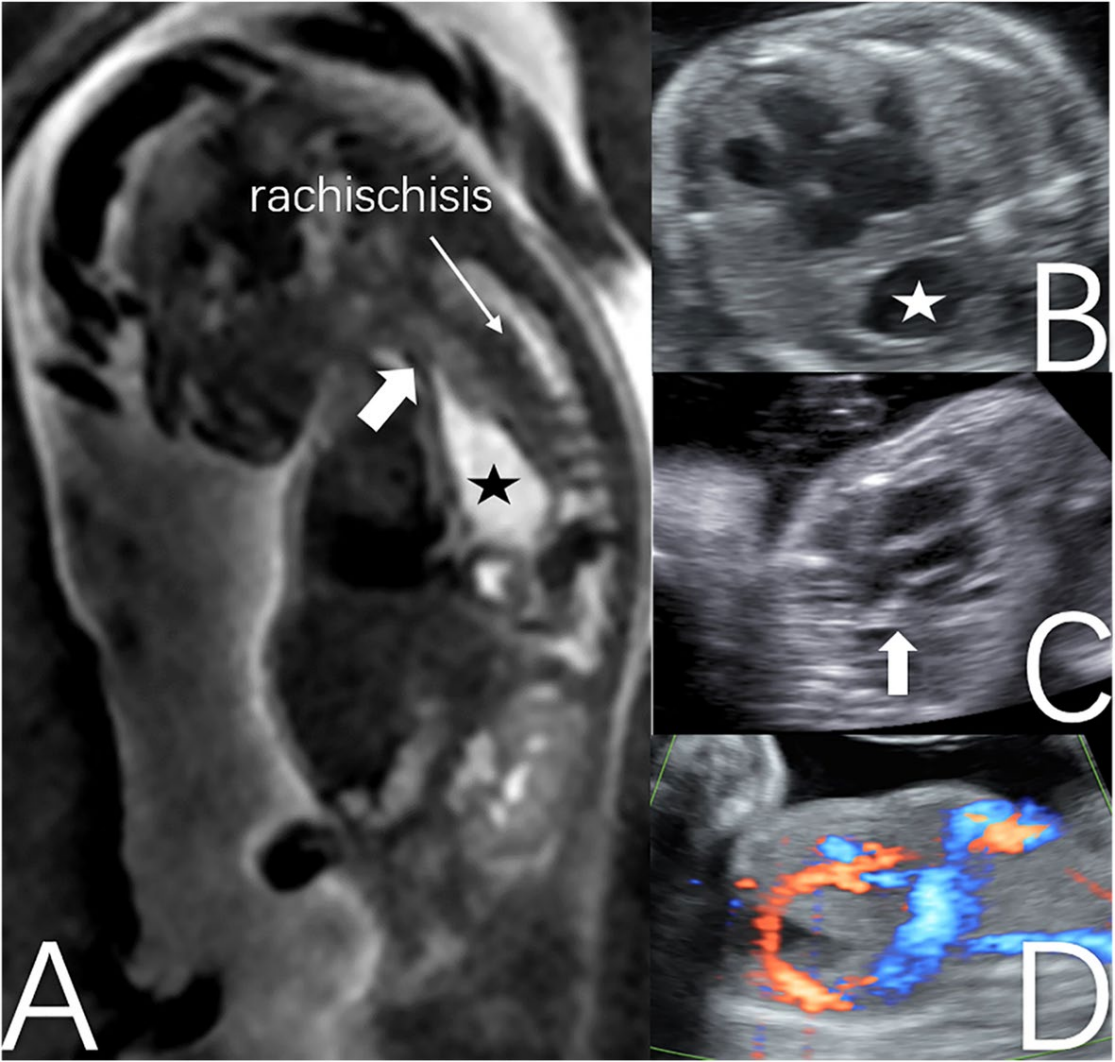
The MR imaging of short oesophagus, intrathoracic stomach, and rachischisis (**A**). The intrathoracic stomach behind the heart (**B**). The oesophagus located at the upper edge of the heart (**C**). Hepatic portal vein passed behind intrathoracic stomach and entered the right atrial (**D**). (Asteroids refer to stomach, thick arrows refer to oesophagus)

Herein, we present a 34-year-old pregnant woman. The patient initially conceived via artificial insemination. However, her pregnancy was subsequently terminated. Next, she had her second artificial insemination and eventually got pregnant. During the first trimester of pregnancy, a nuchal translucency (NT) measuring 1.8 mm was detected. Moreover, the ductus venous was absent, and a cystic mass was detected in the thoracic cavity. The pregnant woman decided to continue with the pregnancy. Routine foetal ultrasonography and re-examination were performed at 16 and 17 weeks of gestation in another hospital, and the diagnoses differed ((the cystic structure in thoracic cavity VS congenital diaphragmatic hernia (CDH)). At 19 week gestation, 2D-US was conducted at our centre, and the following results were obtained (Fig. 1):

- A cystic structure was observed from the pharynx to the diaphragm in the right thoracic cavity. However, there was no gastric bubble in the abdominal cavity.
- No ductus venous was found. The hepatic portal vein passed behind the intrathoracic stomach and entered the right atrium.
- The probability of oesophagobronchial fistula was not ruled out.

MRI was performed at 21-week gestation, and the following results were obtained (Fig. 1):

- A large cystic structure was noted in the right thoracic cavity of the foetus. However, there were no presence of an intra-abdominal stomach and no filling in the intestine. The diaphragm, which is in the lower posterior part of the heart, was not evident, thereby indicating the existence of diaphragmatic hernia.
- There might be a probability of oesophageal atresia with oesophagobronchial fistula.
- Right lung dysplasia with a small volume of right pleural effusion was observed.
- The continuity of the foetal cervical vertebral body and the anterior edge of the spinal canal might be interrupted, indicating the probability of rachischisis. The anterior edge of the foetal cervical vertebrae might be closely connected with the cystic structure.

**Fig. 2:**
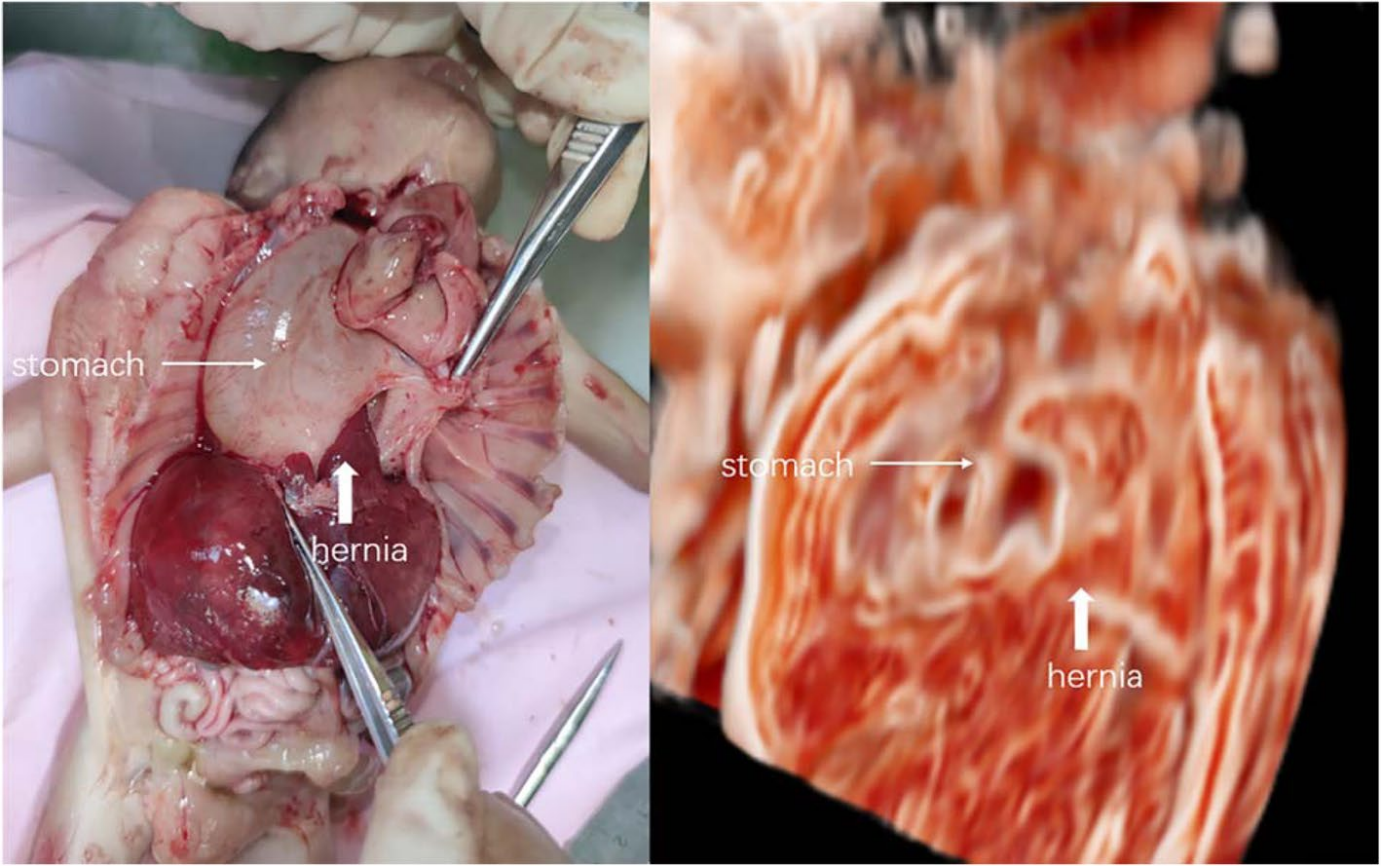
Abdominal organs enter the thoracic cavity, and nearby intrathoracic stomach

**Fig. 3:**
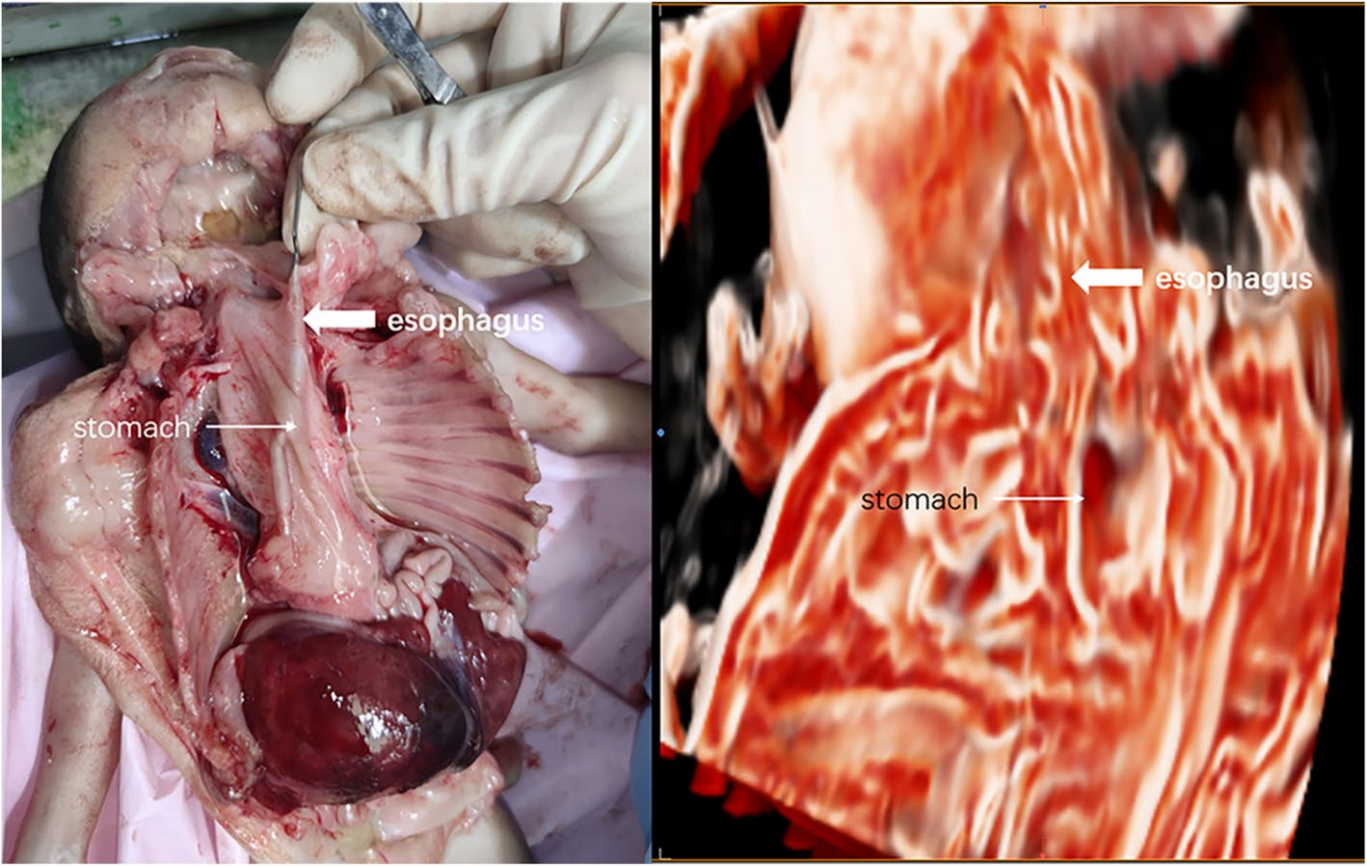
The foetal oesophagus connected with intrathoracic stomach

Three-dimensional US images were obtained at 23-week gestation and were post-processed with Crystal Vue. The following results were obtained:

- The defect in the middle and posterior diaphragm and some abdominal organs partly entered the chest cavity (Fig. 2).
- The oesophagus was short, with a length of 1.5 cm. The end of the oesophagus was connected to the cystic structure, which confirmed the existence of an intrathoracic stomach (Fig. 3).

**Fig. 4:**
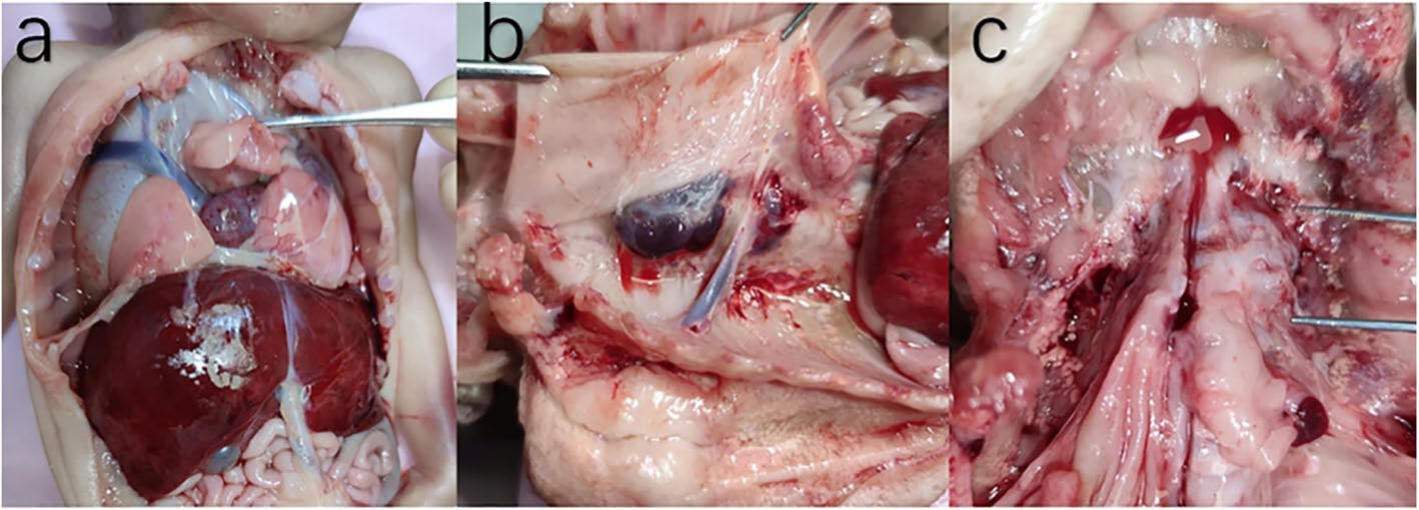
The multiple organs deformations: right lung presented as two lobes, abnormal position of superior vena cava, hepatomegaly (**a**); poly-spleen behind intrathoracic stomach (**b**); hemivertebra and rachischisis (**c**)

The patient terminated the pregnancy, and an autopsy was then performed with the patient’s consent. The autopsy results were as follows:

- The foetus weighed 650 g, and its length was 34 cm. Moreover, short neck and abdominal retraction were noted.
- The cervical vertebra was bent to the left, and the foetal head was inclined to the right shoulder. Vertebral dysplasia was observed at the C2–C6 levels with C2–C4 vertebral body fusion and disorganization, and there was an anterior fissure at the C5–C6 levels with the defect size was 0.4 × 0.3 cm. However, the spinal cord tissue was normal. The defect in the cervical spine was closely connected to the serosa of the transitional area of the oesophagus and cardia.
- The cardia was fixed at the site of cervical spondylolysis in front of the C6 level. The whole stomach was in the right thoracic cavity with a reversed rotation, thereby suggesting a lesser curvature on the left and a greater curvature on the right. After pushing the stomach, a clear yellow liquid flowed out from the nasal cavity.
- On microscopy, the smooth muscle of the stomach was connected to the cartilage.
- The oesophagus was short, with a length of 1.5 cm.
- The duodenum presented with atresia. However, there was no meconium in the intestine.
- There was a defect in the diaphragm at the dorsal side. Parts of the duodenum, pancreas, left and caudate lobes of the liver and whole spleen were in the right chest. The spleen (with poly-spleen malformation) was behind the stomach.
- The mediastinum and the heart were at the left thoracic cavity.
- Right lung dysplasia with bilobate deformity was observed. The superior lobe of the right lung was located at the middle of the chest in front of the mediastinum, and the lower lobe at the right chest.
- The superior vena cava flowed into the right atrium above the interlobular fissure. The main trunk of the hepatic portal vein was pulled into the thoracic cavity and directly flowed into the superior vena cava. No innominate vein was found. The left superior vena cava persistently flowed into the right atrium (Fig. 4).

The foetus was diagnosed with SLS due to the coexistence of brachioesophagus, secondary intrathoracic stomach and vertebral anomalies. Chorionic villus samples were obtained at 13 weeks of gestation. G-banded karyotype analysis was performed, and results did not reveal chromosomal abnormalities. Simultaneously, array comparative genomic hybridization and polymerase chain reaction Gene Scan did not show gene copy number variations. The whole genome sequence of humans was provided using the high-throughput sequencing technology. No pathogenic variants were identified. The likely pathogenic variants were found in MKS transition zone complex subunit 1 (MKS1, chr17:56284466, NM_017777.3: C.1387C > T) and docking protein 7 (DOK7, chr4:3494599, NM_173660.4: C.886C > T). Variants of uncertain significance were detected in microtubule actin crosslinking factor 1 (MACF1, chr1:39888093, NM_012090.4: c.9678G > C), beta 3-glucosyltransferase (B3GLCT, chr13:31789179, NM_194318.3: c.71-9 T > C) and RAB3 GTPase activating non-catalytic protein subunit 2 (RAB3GAP2, chr1:220325081, NM_012414.3: c.3893A > C). Two genetic specialists were consulted, and they confirmed that such variants were not associated with the clinical phenotype.

## Discussion and conclusions

Snakes have an intrathoracic stomach and rachischisis-like spinal vertebrae. In 2008, Michael S. Katz et al. defined SLS as a condition characterized by foetal malformations such as short oesophagus, intrathoracic stomach and vertebral rachischisis. Moreover, in the same year, Alex W.K. Leung et al. reported a foetus presenting with the characteristics of SLS including thoracic hemivertebrae at the T5 level, short oesophagus and intrathoracic stomach. The current study assessed case reports about SLS in the English literature. In total, nine cases were included in our review, and the current case is the 10th.

Table 1 shows the general information about pregnant women and their foetuses. There were two primiparas and five multiparas. Three articles did not include information about the history of pregnancy and childbirth. All multipara women (*n* = 5) had a history of adverse pregnancy. Of 10 foetuses, 5 were male and 5 female. Of five foetuses who underwent surgery, three (60%) died, and two (40%) survived with the help of assistive devices. Moreover, three foetuses died without surgery. In two cases, the pregnancy was terminated. Eight patients were misdiagnosed with CDH and one with pulmonary sequestration. Further, one patient was diagnosed with polyhydramnios and an absent gastric bubble.

**Table 1:** General data of pregnant women and foetuses

Case	Maternal age	History of gestation	Previous diagnosis	Foetal gender	Outcome
case 1 [2]	28	none	hiatal hernia\ pulmonary sequestration\ diaphragmatic hernia	46, XX	death after operation
case 2 [1]	40	G1P0\artificial insemination	polyhydramnios and an absent gastric bubble	46, XX	alive after operation
case 3 [1]	18	none	diaphragmatic hernia \ possible transposition of the great vessels	46, XX	death without operation
case 4 [5]	44	G3P0	median diaphragmatic hernia with intrathoracic stomach	46, XY	death after operation
case 5 [6]	28	none	congenital diaphragmatic hernia	46, XY	death after operation
case 6 [7]	37	G1P0	congenital diaphragmatic hernia	46, XY	death without operation
case 7 [8]	27	G2P0	encephalocele, cystic hygroma and polyhydramnios	46, XX	alive after operation
case 8 [9]	none	G2P0	midline diaphragmatic hernia	46, XX	death without operation
case 9 [3]	35	G3P0	congenital diaphragmatic hernia	46, XY	termination of pregnancy, died
our case	34	G2P0	congenital diaphragmatic hernia\ Cystic structure of thoracic cavity\ esophago-tracheal fistula	46, XY	termination of pregnancy, Died

Table 2 shows data about concomitant deformities. All patients presented with brachioesophagus, intrathoracic stomach and spinal deformity. Nine foetuses had normal karyotypes. However, the genetic results of one patient were unknown. Eight (80%) patients presented with deformities in the intestine, seven (70%) in the spleen, four (40%) in the lungs, four (40%) in the great vessel, three (30%) in the heart, three (30%) in the limbs, three (30%) in the liver, three (30%) in the gallbladder and cystic canal, two (20%) in the stomach outlet and two (20%) in the central nervous system.

**Table 2:** Multiple organs malformation information

Case	Short oesophagus	Intrathoracic stomach	Spinal deformity	Deformity of other organs	Intrathoracic abdominal organs	Karyotype
case 1 [2]	yes	yes	T5 hemivertebra	stomach outlet	part of intestine	normal
case 2 [1]	yes	yes	cervical spondylolysis (C1-C4)	right ulna and radius\ stomach outlet\ spleen	spleen	normal
case 3 [1]	yes	yes	cervical spondylolysis (C2-C5); small T5 vertebral body	right foot\ spleen\ superior vena cava\ heart\ ductus arteriosus\ pulmonary venous\ gut	none	normal
case 4 [5]	yes	yes	cervical scoliosis and fusion	right lung\ umbilical artery\ ductus venosus\ cardiomegaly\Intestinal\ liver\ gallbladder\ colon\ fingers	duodenum\ head of pancreas\ spleen\ first jejunal loop	normal
case 5 [6]	yes	yes	cervical spondylolysis	duodenal	duodenum\ pancreas \spleen	normal
case 6 [7]	yes	yes	cervical hypoplastic and rachischisis and partly fused and disorganization	duodenum\ spleen\ gallbladder\ pancreas	none	normal
case 7 [8]	yes	yes	split notochord malformation	encephalocele, midline localized liver	none	unknown
case 8 [9]	yes	yes	large fissure from cervical to thoracic vertebra	FGR\ ears\ lip\ palmar creases\ bladder\heart \ superior vena cava\ lung\ cystic canal	spleen, part of pancreas	normal
case 9 [3]	yes	yes	severe case of rachischisis from lower cervical to upper thoracic vertebrae	heart position\ lungs	spleen, pancreas, part of duodenum	normal
our case	yes	yes	C2-C6 dysplasia; C2-C4 fusion and disorganization; anterior fissure of C5-C6	right lung\ spleen\ duodenal\ persistent left superior vena cava	part of duodenum, pancreas, part of liver, spleen	normal

The prognosis of SLS is poor, and the survival rate after surgery is 40%. The outcome of the surviving foetuses is poor. Fetoscopic endotracheal occlusion (FETO) can improve the survival rate of foetuses with CDH [10]. Left-sided diaphragmatic hernia is the most common type of CDH, and it may lead to intrathoracic stomach and pulmonary hypoplasia, which is similar to SLS. Intrathoracic stomach compression can impair the development of foetal lungs. FETO might improve the survival of foetuses with SLS. However, compared with CDH, FETO might be less beneficial in the foetus of our report. In CDH, the abdominal organs that herniated into the thoracic cavity are not fixed. Resistance from the hernia sac in CDH is lesser than that from the fixed intrathoracic stomach in our case. No genetic variant associated with the clinical phenotype was detected via G-banded karyotype analysis, array comparative genomic hybridization, polymerase chain reaction Gene Scan and the whole genome sequence of humans. The cause of SLS remains unclear. All multipara women (*n* = 5) had a history of adverse pregnancy. Hence, this factor can be correlated with SLS. In 8 of 10 cases, spinal deformities occurred in the cervicothoracic segment. This phenomenon might be attributed to unknown factors affecting the differentiation of the cervical vertebral body and oesophageal development. Hence, as there is no adhesion and fistula between the oesophagus and trachea, this condition may occur during the embryonic stage, which is after the foregut differentiates into the trachea and oesophagus and before the oesophageal lengthens. A short oesophagus may cause diaphragmatic dysplasia. In the embryonic stage, the dorsal mesentery of the oesophagus develops into the dorsal and median parts of the diaphragm [11]. Based on the current and previous reports, the oesophageal dorsal mesentery does not play a role in the development of diaphragmatic muscle in SLS, resulting in partial dysplasia in the dorsal and median parts of the diaphragm. In this case, the dorsal mesentery of the oesophagus might have fused with the adjacent visceral pleura. Our hypothesis could be validated by the autopsy results. That is, the serosa at the cardia was fixed in front of the C6 level, and the smooth muscle of the stomach was connected to the cartilage. The bilobar malformation of the right lung could possibly be caused by gastric compression in the chest or by failure in the division and development of the right lung bud during the different phases of lung development [12]. Spleen development starts in the 5th week of gestation, and its location changes with the stomach between the 6th and 7th weeks. Asplenia and polysplenia are rare congenital abnormalities of the spleen, 40–70% of them combined with other defects [13]. In our case, the foetal stomach and spleen developed in the thoracic cavity. In CDH, the position and morphology of the spleen and stomach were normal during the embryonic stage. Even if the spleen is in the hernia, it has a normal morphology, and its location is not fixed. In SLS, the spleen had an abnormal morphology, and its location is fixed.

Differential diagnosis

1. The diaphragmatic defect in CDH differs from that in SLS, except for the presence of oesophageal hiatal hernia. In SLS and oesophageal hiatal hernia, the diaphragmatic defects are in the middle and posterior side of the diaphragm. In other CDH types, the diaphragmatic defects are in other locations [14].
2. When an intrathoracic stomach is detected, it is mandatory to exclude CDH. In all CDH types, the abdominal organs that herniated into the thoracic cavity are not fixed. With high thoracic pressure, the herniated organs may partly enter the abdominal cavity [15]. In SLS, the stomach and other organs that should be in the abdominal cavity are fixed in the chest, and the other abdominal organ does not herniate into the thorax.
3. The location and morphology of the spleen in CDH are commonly normal. However, in SLS, it may be located in the thoracic cavity, and deformities including poly-spleen and no-spleen are observed occasionally.

Differential diagnosis of other thoracic diseases

1. SLS is characterized by diaphragmatic dysplasia and intrathoracic diseases caused by an integral diaphragm.
2. The blood vessels of the intrathoracic stomach come from the celiac trunk, the blood vessels of pulmonary sequestration from the aorta and the blood supply of other thoracic diseases from the thoracic vessels [16].
3. The size and location of the stomach are normal in congenital lung lesions.

The length of the oesophagus, integrity of the diaphragm and location of the stomach are important for obtaining a differential diagnosis in SLS with CDH and other thoracic diseases. Since 2D-US remains the standard tool for morphology scan screening, foetal morphological features assessed via 2D-US should be further addressed in detail. Novel 3D-US rendering technique is effective for identifying the perception of depth while preserving context and surface information [17]. Moreover, it can detect the subtle structures of foetuses such as those of the oesophagus, diaphragm and stomach, and the relationships between different structures. Therefore, it has a greater diagnostic value for multiorgan malformations.

## Data Availability

Datasets from this study are available upon reasonable request from the corresponding author.

## Abbreviations

SLS: Serpentine-like syndrome
WGS: Whole genome sequencing
US: Ultrasound
2D-US: Two-dimension ultrasound
3D-US: Three--dimension ultrasound
MR: Magnetic resonance
CDH: Congenital diaphragmatic hernia

## References

[1] Katz MS, Hess DJ, Caty MG, Khan AR, Glick PL (2008). Of snakes and babies: intrathoracic stomach and vertebral rachischisis. A serpentine-like syndrome?. J Pediatr Surg.

[2] Leung AW, Lam HS, Chu WC, Lee KH, Tam YH, Ng PC (2008). Congenital intrathoracic stomach: short esophagus or hiatal hernia?. Neonatology.

[3] Mimura K, Endo M, Matsuoka K, Tomimatsu T, Tazuke Y, Okuyama H, Takeuchi M, Kimura T (2019). Prenatal findings of serpentine-like syndrome with congenital intrathoracic stomach: differential diagnosis from congenital diaphragmatic hernia. J Med Ultrason (2001).

[4] Ddall'asta A, Grisolia G, Nanni M, Volpe N, Schera GBL, Frusca T, Ghi T (2019). Sonographic demonstration of fetal esophagus using three-dimensional ultrasound imaging. Ultrasound Obstet Gynecol.

[5] Deprez FC, Debauche C, Clapuyt P, de Goyet JD (2009). Multiorgan developmental anomalies presenting as a variation of the serpentine-like syndrome: cervical fusion and brachioesophagus with intrathoracic stomach and malposition of duodenopancreas and spleen. J Pediatr Surg.

[6] Nakamura H, Okazaki T, Koga H, Lane GJ, Yamataka A (2012). Congenital brachioesophagus with secondary intrathoracic stomach associated with rachischisis described as "serpentine-like syndrome": a case report and literature review. Pediatr Surg Int.

[7] Dargan D, Mcmorrow A, Bourke TW, Mccallion WA, Verner AM, Lyons J, Mcconnell RS, Lundy CT, Eames NW (2013). Extensive cervical spine and foregut anomaly in 'serpentine syndrome'. Int J Surg Case Rep.

[8] Dorum BA, Korkmaz S, Ozkan H, Koksal N, Bagci O, Yazici Z, Gurpinar AN (2016). Serpentine-like syndrome associated with encephalocele. Clin Dysmorphol.

[9] Beleza-Meireles A, Steenhaut P, Hocq C, Clapuyt P, Bernard P, Debauche C, Sznajer Y (2017). "serpentine-like syndrome"-a very rare multiple malformation syndrome characterised by brachioesophagus and vertebral anomalies. Eur J Med Genet.

[10] Chatterjee D, Ing RJ, Gien J (2020). Update on congenital diaphragmatic hernia. Anesth Analg.

[11] Kaufmann P (2000). The Anatomical Basis of Mouse Development. By MATTHEW H. KAUFMAN and JONATHAN B. L. BARD. (Pp. xii+291; fully illustrated; $99.95; ISBN 0 12 402060 7.) San Diego: Academic Press. 1999. J Anat.

[12] Cotten CM (2017). Pulmonary hypoplasia. Semin Fetal Neonatal Med.

[13] Varga I, Babala J, Kachlik D (2018). Anatomic variations of the spleen: current state of terminology, classification, and embryological background. Surg Radiol Anat.

[14] Kosiński P, Wielgoś M (2017). Congenital diaphragmatic hernia: pathogenesis, prenatal diagnosis and management — literature review. Ginekol Pol.

[15] Kovler ML, Jelin EB. Fetal intervention for congenital diaphragmatic hernia. Semin Pediatr Surg. 2019;28(4). 10.1053/j.sempedsurg.2019.07.001. 10.1053/j.sempedsurg.2019.07.001 31451175

[16] Zobel M, Gologorsky R, Lee H, Vu L (2019). Congenital lung lesions. Semin Pediatr Surg.

[17] Shah H, Al-Memar M, De Bakker B, Fourie H, Lees C, Bourne T (2017). The first-trimester fetal central nervous system: a novel ultrasonographic perspective. Am J Obstet Gynecol.

